# In Their Own Words: A Study on COVID-19-Related Concerns and Perceptions of Risk Among Older University Employees

**DOI:** 10.1093/geroni/igab046.249

**Published:** 2021-12-17

**Authors:** Amanda Sokan, Nicole Yuan, Mariana Felix, Mark Wager, Lisa O'Neill, Zhao Chen

**Affiliations:** 1 University of Arizona, Phoenix, Arizona, United States; 2 University of Arizona, Tucson, Arizona, United States; 3 University of Arizona, Center on Aging, Tucson, Arizona, United States; 4 The University of Arizona, Tucson, Arizona, United States

## Abstract

The purpose of this qualitative study was to examine COVID-19-related concerns and risk perceptions among older employees aged 50+ related to reopening a large state university campus during the COVID-19 pandemic. Recruitment focused on older employees from diverse backgrounds and job classifications. Six focus group interviews, with a total of 24 participants, were conducted using Zoom video conferencing. Interviews were transcribed using Zoom and were double-checked for accuracy. Transcripts were coded and analyzed using ATLAS.ti 9 after establishing inter-rater reliability among two coders. During the campus reopening, older employees reported several concerns and perceptions of risk focused on COVID-19 exposure and transmission to others, individual health and health of other household members, mental health and stress, and job security. Findings were used to inform the development of intervention strategies and resources to promote the health and well-being among older employees during the pandemic.

